# Burnout in Relation to Specific Contributing Factors and Health Outcomes among Nurses: A Systematic Review

**DOI:** 10.3390/ijerph10062214

**Published:** 2013-05-31

**Authors:** Natasha Khamisa, Karl Peltzer, Brian Oldenburg

**Affiliations:** 1 School of Health Sciences, Department of Public Health, Monash South Africa, 144 Peter Road, Roodepoort, Johannesburg 1725, South Africa; 2 Faculty of Medicine, Nursing and Health Sciences, Monash University, Victoria, Melbourne 3800, Australia; E-Mail: brian.oldenburg@monash.edu; 3 Human Science Research Council, 134 Pretorius Street, Pretoria 0002, South Africa; E-Mail: kpeltzer@hsrc.ac.za; 4 University of Limpopo, University Street, Turfloop, Sovenga, Polokwane 0727, South Africa; 5 ASEAN Institute for Health Development, Mahidol University, Salaya 73170, Thailand; 6 Monash Alfred Hospital Campus, Level 3 Burnet Tower, 89 Commercial Road, Melbourne 3004, Australia

**Keywords:** work related stress, burnout, job satisfaction, general health, staff nurses, relationship

## Abstract

Nurses have been found to experience higher levels of stress-related burnout compared to other health care professionals. Despite studies showing that both job satisfaction and burnout are effects of exposure to stressful working environments, leading to poor health among nurses, little is known about the causal nature and direction of these relationships. The aim of this systematic review is to identify published research that has formally investigated relationships between these variables. Six databases (including CINAHL, COCHRANE, EMBASE, MEDLINE, PROQUEST and PsyINFO) were searched for combinations of keywords, a manual search was conducted and an independent reviewer was asked to cross validate all the electronically identified articles. Of the eighty five articles that were identified from these databases, twenty one articles were excluded based on exclusion criteria; hence, a total of seventy articles were included in the study sample. The majority of identified studies exploring two and three way relationships (n = 63) were conducted in developed countries. Existing research includes predominantly cross-sectional studies (n = 68) with only a few longitudinal studies (n = 2); hence, the evidence base for causality is still very limited. Despite minimal availability of research concerning the small number of studies to investigate the relationships between work-related stress, burnout, job satisfaction and the general health of nurses, this review has identified some contradictory evidence for the role of job satisfaction. This emphasizes the need for further research towards understanding causality.

## 1. Introduction

Burnout is typically characterised by emotional exhaustion (depletion of emotional resources and diminution of energy), depersonalization (negative attitudes and feelings as well as insensitivity and a lack of compassion towards service recipients) and a lack of personal accomplishment (negative evaluation of one’s work related to feelings of reduced competence) [1,2]. These three characteristics emphasise the connection between burnout and working with people [3].

Burnout is usually thought of as an individual’s response to prolonged work related stress, which in turn, impacts on job satisfaction and thereafter, can often affect productivity, performance, turnover and wellbeing among health care professionals and other kinds of workers [3]. Health care professionals in general are thought to have a high vulnerability to burnout as a result of experiencing high levels of emotional strain, owing to stressful working environments exacerbated by sick and dying patients to whom they provide care [4]. Nurses in particular however, have been found to experience higher levels of burnout compared to other health care professionals [5,6], owing to the nature of their work [7,8].

High levels of burnout among nurses have often been attributed to prolonged direct personal contact of an emotional nature with a large number of patients [4,9,10]. This, amongst other factors such as prolonged exposure to work related stress as well as low levels of job satisfaction, have also been recognised as factors contributing to high levels of burnout among nurses [11,12]. Burnout in nurses has been shown to lead to emotional exhaustion as well as a loss of compassion for others (depersonalization) and a sense of low personal accomplishment. These experiences can have very significant implications for the health and wellbeing of nurses [13,14,15].

Research has confirmed that prolonged exposure to work related stress is associated with burnout [9], through active interactions between an individual and their working environment. During such interaction, environmental demands exceeding individual resources may be perceived as stressful and result in negative outcomes such as low job satisfaction, burnout and illness [16,17]. In nursing, these demands also include role ambiguity, role conflict, responsibility for others’ lives, work overload, poor relationships at work, inadequate salaries, lack of opportunities for advancement, a lack of personnel, patient care, lack of support, staff issues and overtime [10,18,19].

**Figure 1:**
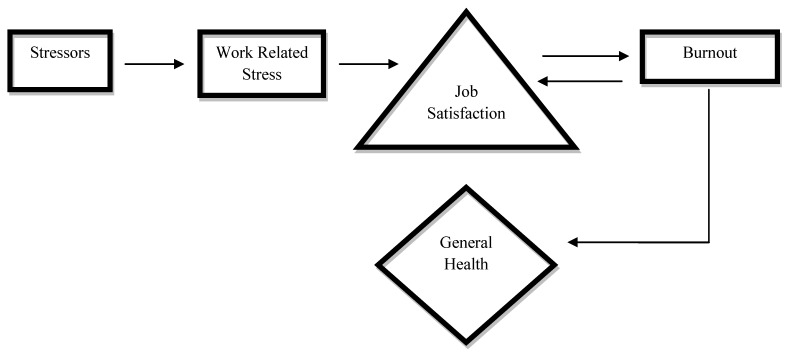
A model illustrating relationships between contributing factors and health outcomes of burnout among nurses.

Limited research has identified studies confirming two and three way relationships between work related stress and job satisfaction [20], work related stress, job satisfaction and burnout [21], as well as work environment and burnout [22] specifically among nurses. However, despite studies showing that both job satisfaction and burnout are effects of exposure to stressful working environments, leading to health consequences [23], the nature and direction of these relationships remains ambiguous (Figure 1).

For the purpose of this review, general health outcomes are specifically defined as being symptoms related to anxiety, depression, somatic symptoms and/or social dysfunction [24].

Although it is already known that nurses experience higher levels of burnout compared to other health care professionals [5,6] and that lack of job satisfaction and burnout result from the effects of exposure to stressful working environments, leading to poor health among nurses [23], little is known about the causal nature and direction of these relationships. Therefore, this systematic review aims to identify those published studies that explore such relationships between work related stress, burnout, job satisfaction and general health, specifically among nurses, while at the same time, also identifying important evidence gaps in the published literature. This can provide a strong foundation for further research in this field as a precursor to conducting controlled evaluations of appropriate intervention strategies.

The review questions are as follows:

- Do existing studies identify the causal nature and direction of relationships between work related stress, burnout, job satisfaction and general health of nurses?
- Do existing studies focus mostly on two and three way relationships between work related stress, burnout, job satisfaction and general health of nurses?

## 2. Methods

### 2.1. Search Strategies

A comprehensive range of search strategies as per the CRD guidelines on EQUATOR were used to identify relevant published studies. Firstly, all of the major public health, psychology and nursing databases were searched for combinations of keywords such as work related stress, burnout, job satisfaction, general health, relationship and nurses. These databases consisted of CINAHL Plus, COCHRANE Library, EMBASE, MEDLINE, PROQUEST and PsycINFO. The second strategy involved a manual search of various journals including the ISRN Nursing, Journal of Nursing Management and Journal of Clinical Nursing using the same combinations of keywords mentioned above. Specific inclusion and exclusion criteria explained below were used to select articles. A third strategy involving an independent reviewer was also used to cross validate all the electronically identified articles. The citation for each identified article was saved using a reference program known as End Note and the full text version was saved in specific folders.

### 2.2. Inclusion Criteria

This review included studies conducted between 1990 and 2012 that: (a) were published in the English language; (b) published only in academic and scholarly journals; (c) were openly accessible and available in full text; (d) were based on empirical studies; (e) measured the relationship between at least two of the variables (work related stress, burnout, job satisfaction and general health); (f) focused on studies specifically consisting of nurses as the sample; and (g) focused on nurses working in various settings (public hospitals, private hospitals, clinics, retirement homes, hospices, mental institutions, prison institutions in urban and rural areas).

### 2.3. Exclusion Criteria

This review excluded studies that: (a) involved insufficient details (such as significance of results/*p*-values) of the identified relationships between work related stress, burnout, job satisfaction and general health; (b) included samples consisting of health professionals in general (doctors, nurses, radiologists, anesthesiologists, social workers); (c) measured different health outcomes beyond the scope of the review (cardiovascular heart disease, diabetes and hypertension). It is believed that exclusion based on the above criteria, allowed for the selection of articles with sufficient information about the method, sample and findings of studies. Selected articles included in this review were analyzed according to their findings and reported in terms of the relationships between work related stress, burnout, job satisfaction and general health of nurses.

## 3. Results

**Figure 2:**
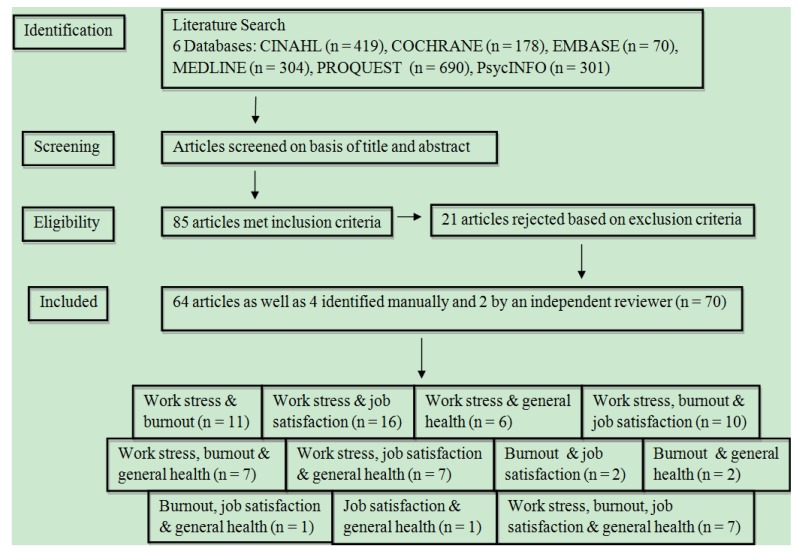
A flow chart describing selection of articles using inclusion and exclusion criteria.

Using the first strategy, a total of eighty five articles meeting the inclusion criteria were electronically identified from six databases. However, following application of the exclusion criteria, twenty one of the eighty five articles were excluded leaving sixty four relevant articles. Four additional articles were identified manually and two by an independent reviewer resulting in a total of 70 articles. This is illustrated below (Figure 2).

Of the 70 identified articles, 64 articles were identified electronically, four articles were identified manually and two articles were identified by an independent reviewer. Of these 70 identified articles, majority were from developed countries (26 European studies, 25 North American studies, 12 Asian studies, four Australian studies, one South African study, one Nigerian study and one East African study).

### 3.1. Work Related Stress and Burnout

Ten articles confirming the relationship between work related stress and burnout were identified. Work environment related stressors such as working place, poor peer relationships, poor nurse patient relationships, lack of professional recognition or reward [25,26,27], feedback clarity and supervisor leadership style [28] were related to one or more burnout dimensions. Work content related stressors such as nursing role, patient care, job demands [25,26,29], job complexity [28], work overload, working overtime [30,31,32], stigma and discrimination while caring for HIV positive patients [29], role conflict, role insufficiency, role ambiguity were also related to burnout [27,30,33]. Nurses who reported inadequate communication with doctors about patients as well as fear of not completing tasks also reported high burnout [34]. A manual search yielded one relevant article, which revealed that burnout (including all three dimensions) is most frequently associated with recurrent night duty among nurses [35].

Further details about the method, sample and findings of identified articles are included in Table 1 below.

**Table 1:** Method, sample and findings of identified articles.

Method	Sample	Findings
Quantitative (questionnaire distribution at conferences and meetings) [25]	132 nurses (132 women & 22 men) working in different wards and clinics [25]	Working place/nursing role was associated with higher burnout among practicing nurses compared to those who had a managerial function (as head nurse, deputy, or mentor) (*t* = 3.2, *p* < 0.01) owing to limited support with complicated treatments, less power, lower status and lack of variation in roles [25]
Quantitative (extensive questionnaire survey) [26]	1,190 registered nurses working in 43 public hospitals [26]	Social context related stressors (lack of professional recognition, professional uncertainty, interpersonal and family conflicts, tension in professional work relationships as well as tensions in nurse-patient relationships) were all significantly associated with emotional exhaustion (β = 0.44, *p* ≤ 0.01), depersonalization (β = 0.26, *p* ≤ 0.01) and personal accomplishment (β = −0.33, *p* ≤ 0.01). Job content related stressors including patient care responsibilities, job demands and role conflict) also had significant relationships with emotional exhaustion (β = 0.22, *p* ≤ 0.01), and personal accomplishment (β = 0.23, *p* ≤ 0.01) but not with depersonalization (β = −0.04, *p* ≥ 0.01) [26]
Quantitative (questionnaire distribution and collection in 2 weeks) [27]	336 nurses (27 male and 309 female) at three hospitals specializing in acute treatment [27]	Emotional exhaustion positively correlated with qualitative workload (β = 0.22, *p* < 0.01), quantitative workload (β = 0.42, *p* < 0.01) and conflict with patients (β = 0.19, *p* < 0.01). Depersonalization was positively related to conflict with other nursing staff (β = 0.28, *p* < 0.01), qualitative workload (β = 0.15, *p* < 0.05), quantitative workload (β = 0.19, *p* < 0.01) and conflict with patients (β = 0.24, *p* < 0.01) while being negatively related to nursing role conflict (β = −0.17, *p* < 0.01). Personal accomplishment was negatively correlated with qualitative workload (β = −0.21, *p* < 0.01) and quantitative workload (β = −0.19, *p* < 0.01) while being positively correlated with nursing role conflict (β = 0.25, *p* < 0.01) [27]
Quantitative (questionnaire distribution with reminders to non responders [28]	492 nurses from long stay wards at 5 psychiatric hospitals [28]	Work environment stressors such as job complexity, feedback/clarity, the level of performance of the patient group and social leadership style explained 16% (adjusted R²) of the variance in emotional exhaustion. Job complexity, feedback/clarity and social leadership style explained 12% of the variance in depersonalization. 11% of the variance in personal accomplishment was explained by feedback/clarity and job complexity [28]
Quantitative and Qualitative (All nurses received questionnaires with 5 being selected to participate in a semi-structured interview) [29]	30 community clinical HIV/AIDS nurse specialists [29]	Significant correlations were found between emotional exhaustion and grief/loss (τ = 0.58, *p* < 0.05), emotional exhaustion and loss tolerance/peer relationship (τ = 0.41, *p* < 0.05), personal accomplishment and social recognition/reward (τ = 0.40, *p* < 0.05). A weak but significant relationship was found between emotional exhaustion and stigma/discrimination (τ = 0.29, *p* < 0.05). Qualitative findings indicated that death of a patient and stigma/grief were related to burnout [29]
Quantitative (questionnaire distribution and completion at 2 time points) [30]	98 nurses attending a post-work course towards a licentiate degree [30]	Amount of variance explained increased (ΔR² = 0.14, *p* < 0.001) when work related stressors were entered into the burnout model. Work overload was the only stressor that significantly predicted emotional exhaustion (β = 0.35, *p* < 0.01). Experience with pain and death significantly predicted depersonalization (β = −0.38, *p* < 0.001) and role ambiguity (β = 0.32, *p* < 0.05) while lack of cohesion (β = 0.24, *p* < 0.05) significantly predicted the lack of personal accomplishment [30]
Quantitative (Questionnaires posted to members of the Association of Nurses in AIDs Care) [31]	445 nurses providing care to people living with HIV/AIDS [31]	Findings confirmed association between perceived workload (hours worked and amount of work) and burnout (r = 0.24, *p* < 0.01). Workload accounted for 5.6% of the variance in burnout [31]
Quantitative (questionnaire packages were mailed to nurses) [32]	574 Australian Nursing Federation members [32]	Generally, working overtime was positively related to higher emotional exhaustion (r = 0.21, *p* < 0.05). Being pressured or expected to work overtime (involuntarily) was related to higher emotional exhaustion (r = 0.41, *p* < 0.05) and depersonalization (r = 0.22, *p* < 0.05); while working unpaid overtime was also associated with higher emotional exhaustion (r = 0.13, *p* < 0.05) [32]
Quantitative (questionnaire distribution by nominated coordinator at each hospital) [33]	495 nurses from three provincial hospitals [33]	Role insufficiency was significantly related to exhaustion (r = 0.38, *p* < 0.05), cynicism (r = 0.39, *p* < 0.05) and professional efficacy (r = 0.28, *p* < .05). Role ambiguity was significantly related to exhaustion (r = 0.20, *p* < 0.05), cynicism (r = 0.28, *p* < 0.05) and professional efficacy (r = 0.27, *p* < 0.05). Role boundary was significantly related to exhaustion (r = 0.29, *p* < 0.05), cynicism (r = 0.34, *p* < 0.05) and professional efficacy (r = 0.21, *p* < 0.05). Responsibility, physical environment, and role overload are all significantly related to exhaustion (r = 0.33, *p* < 0.05, r = 0.31, *p* < 0.05, r = 0.42, *p* < 0.05 respectively) and cynicism (r = 0.28, *p* < 0.05, r = 0.20, *p* < 0.05, r = 0.30, *p* < 0.05 respectively) [33]
Quantitative (questionnaire distribution via the hospital’s internal mail system) [34]	101 registered nurses, employed at a major specialist oncology metropolitan hospital [34]	Significant correlations were found between nursing stressors (lack of support, poor communication with doctors) and emotional exhaustion (r = 0.48, *p* < 0.01) as well as depersonalization (r = 0.34, *p* < 0.01), but not personal accomplishment [34]
Quantitative (questionnaire distribution after receiving consent) [35]	292 nurses working at a state hospital [35]	Doctor/nurse conflict (OR = 3.1; 95% CI, 1.9–6.3), low doctor/nurse ratio (OR = 6.1; 95% CI, 2.5–13.2), inadequate nursing personnel (OR = 2.6; 95% CI, 1.5–5.1) and too frequent night duties (OR = 3.1; 95% CI, 1.7–5.6) were significant predictors of emotional exhaustion. Doctor/nurse conflict (OR = 3.4; 95% CI, 2.2–7.6), low doctor/nurse ratio (OR = 2.4; 95% CI, 1.4– 4.1), and too frequent night duties (OR = 2.4; 95% CI, 1.5– 4.8) significantly predicted depersonalization. High nursing hierarchy (OR = 2.7; 95% CI, 1.5–4.8), poor wages (OR = 2.9; 95% CI, 1.6–5.6) and too frequent night duties (OR = 2.3; 95% CI, 2.3–4.5) significantly predicted reduced personal accomplishment [35]

### 3.2. Work Related Stress and Job Satisfaction

Sixteen articles confirming the relationship between work related stress and job satisfaction were found. Work related stressors including pay, task requirements, well maintained up to date resources [36,37,38], physical work environment [39], autonomy [40,41,42], peer relationships, cohesion, feedback [40,41,43], workload, control over practice [44,45] patient outcomes and supervisor support [36,41] recognition, independence, responsibility, authority [46], meaningfulness of work, nurse centered communication involving humor and clarity [47], role stress [48] as well as overtime [38,41] were related to job satisfaction. It has also been found that the interaction between workload and autonomy best predicts job satisfaction [44]. A common conclusion was that work related stress is significantly related to job satisfaction [49,50] and nurses who experience higher stress levels are less satisfied with their jobs [51]. Further details about the method, sample and findings of identified articles are included in Table 2 below.

**Table 2:** Method, sample and findings of identified articles.

Method	Sample	Findings
Qualitative (interviews, observations and field notes) [36]	8 nurses selected from a local nursing agency [36]	Thematic analysis revealed that nurses were most satisfied with compensation (patient outcomes, compliments, salary, incentives and lessons learned), team spirit (working together and sharing duties), strong support from physicians and advocacy (assisting and supporting new nurses) [36]
Quantitative (questionnaires were sent out with each nurses’ paycheck) [37]	249 nurses employed at a children’s hospital [37]	In general job stress was found to be significantly associated with job satisfaction (r = 0.64, *p* < 0.05). Pay (r = 0.40, *p* < 0.05, r = 0.43, *p* < 0.05), interaction/cohesion (r = 0.44, *p* < 0.05, r = .41, *p* < 0.05) and task requirements (r = 0.53, *p* < 0.05, r = 0.67, *p* < 0.05) were significantly associated with both job stress and job satisfaction respectively [37]
Quantitative (questionnaires were mailed to nurses) [38]	944 RN’s working in rural and remote hospital settings [38]	Workplace stressors explained 32% of the variance in job satisfaction. Having available, well maintained and up-to-date equipment and supplies was highly related to job satisfaction, accounting for 17% of the total variance. Greater scheduling and shift satisfaction (no overtime) as well as lower psychological job demands (fewer time constraints, less excessive workloads) were strong predictors of job satisfaction (accounting for 12% of the variance) [38]
Quantitative (survey packets with instructions were placed in staff mailboxes) [39]	116 medical-surgical nurses working in acute-care settings [39]	Only one environmental factor, noise, was significantly associated with perceived stress (r = −0.18*, p* = 0.05). Perceived stress was directly related to job satisfaction (r = 0.55, *p* = 0.00) [39]
Quantitative (survey distribution via the hospital’s internal mail) [40]	135 nurses employed in a 170 bed hospital [40]	Work content stressors including variety, autonomy, task identity and feedback are all strongly correlated with job satisfaction (r = 0.35–0.50, *p* < 0.001). Work environment stressors including collaboration with medical staff and cohesion among nurses are also strongly correlated with job satisfaction (r = 0.37–0.45, *p* < 0.001). Job satisfaction was mostly predicted by variety, feedback and collaboration with medical staff (r = 0.55, R² = 0.30) [40]
Quantitative (E-mails containing a $5 e-mail gift certificate and a web link to the survey instrument were sent. Reminder e-mails were sent to non responders) [41]	362 registerednurses in a large metropolitan hospital [41]	Job satisfaction was positively and significantly correlated with physical work environment (r = 0.26, *p* < 0.01). Significant positive predictors of job satisfaction from the baseline model were autonomy (β = 0.09, *p* < 0.05), supervisor support (β = 0.05, *p* < 0.05), workgroup cohesion (β = 0.09, *p* < 0.05), working in a unit other than the intensive care unit (β = 0.67, *p* < 0.05), working in a step-down unit or general medical surgical unit (β = 0.31, *p* < 0.05), and number of hours of voluntary overtime worked in a typical work week (β = 0.05, *p* < 0.05). A negative significant predictor was working a 12-hour shift (β = −0.83, *p* < 0.05) [41]
Quantitative (questionnaire distribution through the nurse manager of each unit) [42]	431 critical care nurses, all of whom were RN’s working at 16 different hospitals [42]	Professional autonomy had a moderate positive correlation with reported role conflict and role ambiguity (r = 0.33, *p* < 0.001). A positive moderate correlation between professional autonomy and job satisfaction was found (r = 0.33, *p* < 0.001) [42]
Quantitative (anonymous questionnaire distribution) [43]	117 Registered Nurses (77 Army RNs – 40 Civilian RNs) [43]	Work related stress was inversely correlated with job satisfaction for both civilian (r = −0.32, *p* < 0.05) and army (r = −0.23, *p* < 0.05) nurses. Army nurses were most stressed and least satisfied by their working relations with colleagues (r = −0.40, *p* < 0.01), while civilian nurses were most stressed and least satisfied with their physical working environments (r = 0.32, *p* < 0.05) [43]
Quantitative (participants were invited by e-mail to attend a one-day event where they completed surveys) [44]	271 public health nurses [44]	Control-over-practice (*x*² = 7.22, *p* = 0.01; OR = 1.01, 95% CI 1.00–1.02) and workload (*x*² = 15.04, *p* < 0.01; OR = 0.90, 95% CI 0.86–0.95) significantly predicted job satisfaction. The strongest association was found between workload and job satisfaction, whereby a one-unit increase on the work overload scale decreased the odds of job satisfaction by nearly 10%. The interaction between autonomy and workload was a significant predictor of job satisfaction (*x*² = 15.87, *p* < 0.01) [44]
Quantitative (voluntary completion of standardized questionnaires) [45]	129 qualified nurses [45]	Results showed that workload was the highest perceived stressor in the nurses’ working environment (M = 1.61, SD ± 0.88). Nursing stress was found to be negatively and significantly correlated with job satisfaction (r = −0.22, *p* < 0.05). Nurse stress predictor variables combined accounted for 17% of the variance in job satisfaction (R² = 0.17, F (3, 123) = 8.9, *p* < 0.001) [45]
Quantitative (distribution of questionnaire packets) [46]	140 registered nurses from medical-surgical, management and home health nursing specialties [46]	There was a significantly positive correlation between job satisfaction and perceived autonomy (r = 0.538, *p* < 0.05) [46]
Quantitative (surveys were made available in each unit and were also distributed to nurses during unit meetings with incentives) [47]	205 nurses employed at a at a large women andchildren’s hospital [47]	Nurses’ perceptions of physicians’ nurse centered communication was significantly related to job satisfaction (*r* = 0.23, *p* = 0.002). Physicians’ nurse centered communication behaviors examined as predictors of nurses’ reported job satisfaction revealed a significant model (F (5, 160) = 3.86, R² = 0.11, *p* = 0.003, with humor and clarity being the most significant predictors of job satisfaction). Work environment, meaningfulness of work, and stress also significantly predicted job satisfaction in another model (F (7, 188) = 27.40, R² = 0.51, *p* = 0.001) [47]
Quantitative (anonymous questionnaire distribution and collection) [48]	532 nurses with job rotation experience [48]	Structural equation modeling revealed a negative relationship between role stress and job satisfaction (γ = 0.52, *p* < 0.01) [48]
Quantitative (survey distribution by nurse managers. Follow up surveys were redistributed after 2 weeks to boost response rate) [49]	287 registered nurses employed in state prison health care facilities [49]	The nursing stress score was the strongest explanatory variable, accounting for 30.3% of the variance in job satisfaction. An inverse relationship between nursing stress and job satisfaction was confirmed (β = −0.55, *p* < 0.01) [49]
Quantitative (questionnaire distribution by graduate students and administrative staff to nurses’ onsite mailboxes) [50]	464 RNs employed in five acute care hospitals [50]	Work related stress (including personal stressors (r = −0.11, *p* < 0.05) as well as situational stressors (r = −0.30, *p* < 0.05)) were negatively correlated with job satisfaction. Regression analysis further confirmed that work related stress (personal stressors *(R*² = 0.29, *p* < 0.05) as well as situational stressors (*R*² = 0.29, *p* < 0.05)) is a significant predictor of job satisfaction [50]
Quantitative (questionnaire distribution by nurse administrators)	285 nurses from six hospitals	The strongest association was found between job related stress and job satisfaction, which were inversely related (r_s_ = −0.331, *p* < 0.05). It was concluded that nurses who experience higher stress levels are less satisfied with their jobs.

### 3.3. Work Related Stress and General Health

Six articles confirmed the relationship between work related stress and general health of nurses. The frequency of exposure to stressful situations including emotionally provoking tasks and a lack of social support from peers were related to psychosomatic health complaints [52]. Nurses with work overload and negatively perceived health status reported higher occurrence of headaches [53]. Furthermore, high job demands, low job control and lack of social support at work were related to mental distress even after controlling for age, smoking, alcohol consumption and physical activity [54]. Other work stressors related to physical and mental health include physician conflict and nurse conflict, negative patient outcomes, treatment uncertainty and inadequate preparation [55]. In general, work related stress is negatively related to psychological wellbeing [56] and poor health [57] among nurses. Further details about the method, sample and findings of identified articles are included in Table 3 below.

**Table 3:** Method, sample and findings of identified articles.

Method	Sample	Findings
Quantitative (distribution of self administered questionnaires) [52]	420 registered nurses and student nurses from public hospitals [52]	The frequency of stressful situations and emotionally provoking problems as well as the lack of social support from peers were the only factors significantly associated with psychosomatic health complaints among registered nurses (R² = 0.11, *p* < 0.01) and student nurses (R² = 0.06, *p* < 0.05), after controlling for other variables [52]
Quantitative and qualitative (distribution of questionnaires and interviews by a neurologist) [53]	779 nursing staff at a tertiary medical center [53]	Work overload (M = 3.32, SD ± 0.74, *p* < 0.001) and health status (M = 2, SD ± 1.16, *p* < 0.001) were the most significant stressors among headache sufferers [53]
Quantitative (questionnaire distribution at an event) [54]	372 community nurses [54]	High job demands (OR = 2.15; 95% CI, 1.07–4.30), low job control (OR = 1.22; 95% CI, 0.64–2.31) and job strain/low social support at work (OR = 3.78; 95% CI, 2.08–6.87) were related to mental distress. In conclusion, mental distress among the nurses is associated with occupational stress elicited by adverse psychosocial job characteristics [54]
Quantitative (questionnaire packets distributed by head nurse for each unit) [55]	480 hospital nurses from five hospitals in three major cities [55]	The most frequently occurring workplace stressor was workload (M = 9.18, SD ± 3.93). Work place stressors including workload (r = −0.21, *p* < 0.01, r = −0.30, *p* < 0.01), physician conflict (r = −0.24, *p* < 0.01, r = −0.25, *p* < 0.01), death/dying (r = −0.18, *p* < 0.01, r = −0.17, *p* < 0.01), nurse conflict (r = −0.27, *p* < 0.01, r = −0.28, *p* < 0.01), lack of support (r = −0.11, *p* < 0.01, r = −0.14, *p* < 0.01), inadequate preparation (r = −0.17, *p* < 0.01, r = −0.23, *p* < 0.01) and treatment uncertainty (r = −0.25, *p* < 0.01, r = −0.26, *p* < 0.01) were all significantly correlated with physical and mental health respectively. Work place stress is related to physical and mental health [55]
Quantitative (questionnaire distribution by principal nursing officers in each unit) [56]	1,043 nurses of different grades/ranks/departments [56]	Work stress was found to be negatively related to psychological well-being of the nurses, with stronger effects on anxiety and depression (r = −0.44, *p* < 0.001) [56]
Quantitative (online surveys with email reminders to non responders) [57]	3,132 registered nurses from five multi-state settings [57]	Perceived work stress lev­els was confirmed as a strong predictor of poor health among nurses (OR = 1.09; 95% CI, 1.05–1.13) [57]

### 3.4. Work Related Stress, Burnout and Job Satisfaction

Nine articles confirmed the relationship between work related stress, burnout and job satisfaction. Nurses providing direct care while working in poor environments report higher burnout and lower job satisfaction [58]. It has also been found that improving working environments reduced job dissatisfaction and burnout among nurses [59]. Poor relations with physicians, difficulty meeting patients’ needs, high workload and low job satisfaction are all related to burnout [60]. Nurse staffing was also found to be related to job satisfaction and burnout [61], with increased patient to nurse ratios relating to higher burnout and lower job satisfaction [62] following an increase in the ratio by one patient per nurse [7].

Although work related stressors including nurse physician relationships, management styles and organizational support were found to be related to burnout and job satisfaction [63], further analysis indicated that work related stress is linked to job satisfaction through burnout [64]. 

These findings suggest that burnout plays a mediating role in the relationship between work related stress and job satisfaction. Furthermore, work related stress and burnout were not only associated with job satisfaction, but were strongly predictive [65].

A manual search led to the identification of an additional article confirming that work related stress, burnout and job satisfaction among nurses are significantly related [66]. Further details about the method, sample and findings of identified articles are included in Table 4 below.

**Table 4:** Method, sample and findings of identified articles.

Method	Sample	Findings
Quantitative (nurses were sent surveys at their home mailing address) [58]	95,499 nurses from 614 hospitals in four states [58]	Nurses providing direct care for patients reported higher burnout (94%) and job dissatisfaction (64%). A third of nurses working in poor environments were dissatisfied with their jobs. Nurses who were satisfied with their jobs were twice as high for those working in better environments. It was concluded that nursing roles and working environments affect burnout and job satisfaction among nurses [58]
Quantitative (Surveys were delivered to nurses by nurse managers) [59]	1,104 bedside nurses in 89 medical, surgical and intensive care units at 21 hospitals [59]	Improving the work environments of nurses (from poor to better) was associated with a 50% decrease in job dissatisfaction and a 33% decrease in burnout. The chances of higher burnout and job dissatisfaction were lower among nurses working in good environments than those working in poor environments, by OR = 0.67 and 0.50, respectively. Nurses working in poor environments were 1.5 and 2 times more likely than those working in good environments to experience burnout and job dissatisfaction [59]
Quantitative (the questionnaires were hand delivered to participants and collected within a week) [60]	60 nurses from 3 hospitals [60]	Non satisfactory relations with physicians (M = 30.2, SD ± 6.6, M = 10.8, SD ± 4.8, M = 25.9, SD ± 10) and high difficulty in meeting patient care needs (M = 32.8, SD ± 6, M = 12.2, SD ± 5.1, M = 25.3, SD ± 11.7) as well as low work satisfaction (M = 27.5, SD ± 8, M = 9.3, SD ± 4.5, M = 28.1, SD ± 10.6) were all significantly associated with higher emotional exhaustion, and depersonalization as well as low personal accomplishment respectively. High nursing workload (M = 17.2, SD ± 7.1, M = 35.3, SD ± 8.2) was associated with higher emotional exhaustion and depersonalization respectively [60]
Quantitative (questionnaire distribution and return in sealed envelopes) [61]	1,365 nurses from 65 intensive care units at 22 hospitals [61]	Perceived adequate staffing was related to decreases in the odds of dissatisfaction (OR = 0.30; 95% CI, 0.23–0.40) and burnout (OR = 0.50; 95% CI, 0.34–0.73) [61]
Quantitative (questionnaires were distributed through the hospitals internal mail systems [62]	5,006 English nurses and 3773 Scottish nurses [62]	Significant relationships were confirmed between nurse staffing (nurse to patient ratio) and burnout (odds ratios for burnout increased from 0.57 to 0.67 to 0.80 to 1.00 as the number of patients a nurse was responsible for increased from 0–4 to 5–8 to 9–12 to 13 or greater). The relationship between nurse staffing and job dissatisfaction was also significant (OR = 0.81; 95% CI, 0.71–0.93) [62]
Quantitative (nurses were invited to voluntarily complete questionnaires distributed by an assigned person) [63]	401 staff nurses across 31 units in two hospitals [63]	The improved model confirmed the mediating role of burnout (depersonalization and personal accomplishment) in the relationship between nurse practice environment related stress (nurse-physician relationship, nurse management, hospital management and organizational support,) and job outcomes (including job satisfaction) (*x*² = 548.1; d.f. = 313; *p* < 0.001; CFI = 0.906; IFI = 0.903; RMSEA = 043) [63]
Quantitative (nurses were invited to voluntarily complete questionnaires distributed by an assigned person) [64]	155 medical, surgical and surgical intensive care unit nurses across 13 units in three hospitals [64]	Nurse–physician relations had a significant positive association with nurse job satisfaction (OR = 7.7; 95% CI, 2.6–22.7) and personal accomplishment (OR = 3.5, S.E. ± 0.8), nurse management at the unit level had a significant positive association with the nurse job satisfaction (OR = 3.6; 95% CI, 1.3–10) and personal accomplishment (OR = 2.7, S.E. ± 0.1.1), hospital management and organizational support had a significant positive association with personal accomplishment (OR = 2.1, S.E. ± 1). Nurse–physician relations (OR = −3.9, S.E. ± 1.2) and nurse management (OR = −3.6, S.E. ± 1.6) had a significant negative association with emotional exhaustion, while hospital management and organizational support had a significant negative association with depersonalization (OR = −2.0, S.E. ± 0.8) [64]
Quantitative (nurses were invited to voluntarily complete questionnaires) [65]	546 staff nurses from 42 units in four hospitals [65]	Emotional exhaustion is the strongest predictor of job satisfaction (OR = 0.89, 95% CI 0.85–0.94). Positive ratings on the nurse work practice environment dimensions including nurse-physician relations (Slope = −4, SE ± 0.7, Slope = −1.3, SE ± .4, Slope = 2.2, SE ± 0.5), nurse management (Slope = −8.5, SE ± 1.2, Slope = −3.1, SE ± 0.6, Slope = 4.32, SE ± 0.8) as well as hospital management and organizational support (Slope = −9.5, SE ± 1.1, Slope = −3.9, SE ± 0.6, Slope = 4.7, SE ± 0.8) were significantly correlated with lower emotional exhaustion and depersonalization as well as high personal accomplishment respectively. Hospital management and organizational support is significantly associated with job satisfaction (OR = 10.7, 95% CI 3.1–37) [65]
Quantitative (fieldworkers appointed by hospital management for private hospitals and by the affiliated university for public hospitals were trained to distribute and collect questionnaires) [66]	935 registered nurses working in critical care units of selected private and public hospitals [66]	Significant correlations were found for all the subscales of the practice environment (including nurse manager leadership, ability and support, nurse physician relations, staffing and resource adequacy, nurse participation in hospital affairs) with job satisfaction (rs = 0.30 to .65, *p* < 0.01) and burnout (rs = −0.41 to 0.26, *p* < 0.01). Job satisfaction was also significantly associated with burnout (rs = −0.46 to 0.23, *p* < 0.01) [66]
Quantitative (surveys were mailed to nurses who were members of the Board of Nursing) [7]	10,184 staff nurses providing adult acute care at 210 general hospitals [7]	An increase of one patient per nurse was found to increase burnout by 1.23 (95% CI, 1.13–1.34) and job dissatisfaction by 1.15 (95% CI, 1.07–1.25) confirming an association between these variables. Nurses working in hospitals with 1:8 patient ratios were found to be 2.29 times more likely to experience burnout and 1.75 times more likely to be dissatisfied with their jobs. Lower staffing increases the likelihood of nurses experiencing burnout and job dissatisfaction [7]

### 3.5. Work Related Stress, Burnout and General Health

Six articles confirmed the relationship between work related stress, burnout and general health. Anxiety, depression and somatization are linked to work related stress and burnout [67]. Specific stressors such as higher physical and emotional demands [68] as well as work overload, role stress, hostility with physicians and patients are directly and indirectly related to burnout and psychosomatic complaints [69]. In another study, physical tiredness, working with demanding patients, losing a patient, lack of free time and burnout were also found to be related [70]. Further analysis indicated that burnout plays an intervening role in the relationship between work related stress and health [71]. This was supported, in that, work related stress has been found to be indirectly related to burnout, which was directly related to the health of nurses [72].

Additionally, an article identified by an independent reviewer confirmed that work related stress is significantly related to burnout and mental health [73]. Further details about the method, sample and findings of identified articles are included in Table 5 below.

**Table 5:** Method, sample and findings of identified articles.

Method	Sample	Findings
Quantitative (distribution of survey packets by head nurses/charge nurses) [67]	237 paid staff nurses employed on 18 units in 7 hospitals [67]	More health complaints (anxiety, depression and somatization) were associated with higher work related stress and emotional exhaustion (rs = 0.21 to .42, *p* < 0.001). Work related stress, burnout and health are related [67]
Quantitative (questionnaires were sent to nurses’ home address) [68]	69 nurses from anursing home [68]	High physical demands had adverse effects on physical complaints (β = 0.2, SE ± 0.1) and emotional demands affected emotional exhaustion (β = 0.4, SE ± 0.1) [68]
Quantitative (self reported questionnaire distribution) [69]	1,636 unionized registered nurses (RNs) working in the public health care sector [69]	Demands including overload (*γ* = 0.57, *p* < 0.001), role stress (*γ* = 0.08, *p* < 0.05), hostility with physicians (*γ* = 0.12, *p* < 0.001) and hostility with patients (*γ* = 0.11, *p* < 0.01) are the most significantly important determinants of emotional exhaustion which indirectly affect depersonalization via emotional exhaustion (*γ* = 0.36, *p* < 0.001). Emotional exhaustion (*γ* = 0.71, *p* < 0.001) and depersonalization (*γ* = 0.22, *p* < 0.001) are significantly associated with psychosomatic complaints [69]
Quantitative (All of the centers were sent questionnaires for each one of their nurses) [70]	229 professional nurses from medical centers [70]	High emotional exhaustion was found to be directly associated with physical tiredness (OR = 2.01; 95% CI, 1.12–3.61) and health (OR = 1.47; 95% CI, 1.32–1.63). High depersonalization was found to be associated with health (OR = 1.17, 95% CI 1.07–1.28). Low personal accomplishment was found to be inversely related to losing a patient (OR = 0.46; 95% CI, 0.22–0.97) and lack of free time (OR = 0.43, 95% CI, 0.20–0.93). Physical tiredness and working with demanding patients are associated with burnout. Burnout is associated with poor health [70]
Quantitative (questionnaires were sent to nurses) [71]	297 nurses at a large university hospital [71]	Nursing stress was directly associated with burnout as well as health (affective and physical symptoms), whereby nursing stress predicted burnout which predicted affect and physical symptoms (*x*² = (3, *n* = 259) = 19.07 (RMSR = 0.05, CFI = 0.92). Burnout was confirmed as an intervening variable between work stress and affective and physical symptomatology (*x*² = (1, *n* = 259) = 5.45 (RMSR = 0.01, CFI = 0.98) [71]
Quantitative (questionnaire distribution by nurse managers) [72]	126 registered nurses were recruited from area hospitals [72]	Emotional exhaustion (R^2^ = −0.407; *p* < 0.0001) and depersonalization (R^2^ = −0.034; *p* < 0.05) were inversely predictive of health outcomes whereas personal accomplishment (R^2^ = 0.03; *p* < 0.05) was positively predictive of health outcomes. Work stress is indirectly related to burnout (through mediation by hardiness) and burnout is directly related to health outcomes [72]
Quantitative (questionnaire distribution followed by reminders) [73]	1,891 nurses from 6 acute care hospitals [73]	Work stress was significantly associated with burnout (OR = 5.77; 95% CI, 3.92–8.5) and mental health (OR = 2.34; 95% CI, 1.62–3.36) [73]

### 3.6. Work Related Stress, Job Satisfaction and General Health

Six articles confirmed the relationship between work related stress, job satisfaction and general health. Work related stressors including job complexity, feedback/clarity, leadership styles, opportunities for promotion and growth, autonomy, workload [74,75,76], relations with the head nurse, peers and physicians, job conflict, cooperation, expectations and demands, development and motivation are related to job satisfaction and health complaints [77,78]. Contrary to this, other findings suggest that higher stress levels among nurses were associated with more health complaints but not with job satisfaction [79].

An additional article identified by an independent reviewer revealed that work related stressors are associated with job satisfaction and psychosomatic complaints among nurses [80]. Further details about the method, sample and findings of identified articles are included in Table 6 below.

**Table 6:** Method, sample and findings of identified articles.

Method	Sample	Findings
Quantitative (questionnaire distribution to nurses) [74]	475 senior nurses [74]	Stressors accounted for the largest portion of the variance explaining job satisfaction (career stress = 22% and organizational stress = 3%). Job stress was found to be significantly predictive of job satisfaction (F (6, 468) = 31.8, *p* < 0.001). Only stress associated with workload was found to be a predictor of mental health (accounting for 4% of the variance) [74]
Quantitative (questionnaire distribution to nurses) [75]	561 trained staff nurses from 16 randomly chosen hospitals [75]	Various work dimensions such as job complexity, feedback/clarity, work pressure, autonomy, promotion/growth as well as supervisors’ leadership style are related to job satisfaction (r = 0.18–0.61, *p* < 0.01) and health complaints (r = 0.20–0.34, *p* < 0.01). 59% and 20% of variance in job satisfaction and health complaints is explained by the selected predictors (work dimensions) [75]
Quantitative (questionnaire distribution to nurses) [74]	475 senior nurses [74]	Stressors accounted for the largest portion of the variance explaining job satisfaction (career stress = 22% and organizational stress = 3%). Job stress was found to be significantly predictive of job satisfaction (F (6, 468) = 31.8, *p* < 0.001). Only stress associated with workload was found to be a predictor of mental health (accounting for 4% of the variance) [74]
Quantitative (questionnaire distribution to nurses) [75]	56l trained staff nurses from 16 randomly chosen hospitals [75]	Various work dimensions such as job complexity, feedback/clarity, work pressure, autonomy, promotion/growth as well as supervisors’ leadership style are related to job satisfaction (r = 0.18–0.61, *p* < 0.01) and health complaints (r = 0.20–0.34, *p* < 0.01). 59% and 20% of variance in job satisfaction and health complaints is explained by the selected predictors (work dimensions) [75]
Quantitative (following invitation and awareness questionnaires were distributed) [76]	155 nurses from nine units in two general hospitals [76]	Autonomy and workload are significantly associated with job satisfaction (r = 0.46, *p* < 0.01 and r = −0.33, *p* < 0.01, respectively) and health complaints (r = −0.17, *p* < 0.05 and r = 0.25, *p* < 0.01, respectively). The correlation between complexity of care and job satisfaction was no longer significant (*p* = 0.38) when workload was corrected for. Workload mediates the relationship between complexity and job satisfaction [76]
(Quantitative (questionnaire distribution for completion at own convenience) [77]	376 female hospital nurses working full time at an urban university teaching hospital [77]	In descending order, perceived relations with the head nurse (β = 0.24, *p* ≤ 0.001), job conflict (β = −0.19, *p* ≤ 0.001), relations with coworkers (β = 0.17, *p* ≤ 0.01), relations with physicians (β = 0.15, *p* ≤ 0.01), and other units/departments (β = 0.13, *p* ≤ 0.01) were significant predictors of job satisfaction. Job conflict (β = 0.12, *p* ≤ 0.05), along with the relations with the head nurse (β = −0.12, *p* ≤ 0.05) and physicians (β = 0.09, *p* ≤ 0.05), were predictors of psychological distress. The relations with the head nurse and physicians as well as job conflict, were predictors of both satisfaction and health [77]
Quantitative (questionnaire distribution) [78]	299 staff working in different forms of elderly care [78]	Stressors including workload, cooperation, age, expectations and demands, personal development and internal motivation explained 41% of the variance in perceived stress symptoms. Job satisfaction was positively and significantly associated with perceived stress symptoms including sleep disturbance, depression, headaches and stomach disorders. This model was significant (F(6/280) = 32.54, *p* < 0.001) [78]
Quantitative (self administered questionnaire distribution) [79]	218 female nurses from public hospitals [79]	Nurses with the highest level of stress reported significantly higher frequency of tension headache (32.4%, *p* < 0.001), back-pain (30.1%, *p* < 0.05), sleeping problems (37%, *p* < 0.001), chronic fatigue (59.5%, *p* < 0.001), stomach acidity (31.5%, *p* < 0.01) and palpitations (32.4%, *p* < 0.01). The frequency of psychosomatic symptoms is an indicator of nurse related stress. No relationship was confirmed between job satisfaction and stress [79]
Quantitative (distribution of self administered structured surveys) [80]	254 nurses working in 15 emergency departments of general hospitals [80]	Work-time demands were found to be important determinants of psychosomatic complaints (β = −0.31, *p* < 0.001) and fatigue (β = −0.21, *p* < 0.01) in emergency nurses. Decision authority (β = 0.138, *p* < 0.05), skill discretion (β = 0.17, *p* < 0.01), perceived reward (β = 0.25, *p* < 0.001) and social support by colleagues (β = 0.16, *p* < 0.01) were found to be strong determinants of job satisfaction. Work related stress explained 21% of the variance in psychosomatic complaints and 34% variance in job satisfaction [80]

### 3.7. Burnout and Job Satisfaction

Only one article confirming the relationship between burnout and job satisfaction was identified. It has been found that a two factor model including burnout and job satisfaction was a better fit providing evidence of a negative association between job satisfaction (particularly with supervisors and coworkers) and burnout [81].

Following a manual search, an additional article confirmed that job satisfaction is a significant predictor of burnout among nurses [11]. Further details about the method, sample and findings of identified articles are included in Table 7 below.

**Table 7:** Method, sample and findings of identified articles.

Method	Sample	Findings
Quantitative (questionnaire distribution by administrative officer) [81]	248 nurses from five hospitals [81]	Satisfaction with supervisors and coworkers was significantly negatively associated with emotional exhaustion (r = −0.50, *p* < 0.01 and r = −0.34, *p* < 0.01, respectively) and depersonalization (r = −0.41, *p* < 0.01 and r = −0.29, *p* < 0.01, respectively) while being positively correlated with personal accomplishment (r = 0.19, *p* < 0.01 and r = 0.19, *p* < 0.01, respectively). This two-factor model compared to the single-factor model was a better fit (Δχ² (1) = 572.533, *p* < 0.001) [81]
Quantitative (questionnaire distribution in a quiet room within the hospital) [11]	203 employed nurses [11]	Through path analyses, it was found that job satisfaction had a direct negative effect on emotional exhaustion (−0.97, *p* < 0.01) and on depersonalization through emotional exhaustion (−0.58, *p* < 0.01). Job satisfaction is a significant predictor of burnout in nurses [11]

### 3.8. Burnout and General Health

Two articles revealing a weak but significant relationship between burnout and depression were identified [82,83]. Further details about the method, sample and findings of identified articles are included in Table 8 below.

**Table 8:** Method, sample and findings of identified articles.

Method	Sample	Findings
Quantitative (anonymous distribution of self reported questionnaires) [82]	368 members of the nursing staff [82]	A weak but significant relationship between burnout and depression was found (χ² (3) = 12.093, *p* < 0.01) Younger nurses were found to suffer from burnout and depression (χ² (3) = 13.337, *p* > 0.01), more than elderly nurses (χ²(3) = 5.685, *p* < 0.01) [82]
Quantitative (questionnaire distribution and collection in one sitting) [83]	17 male and 62 female nurses in general internal medicine, general surgery and respiratory medical wards [83]	Depression was correlated with burnout to a lesser degree (r = −0.38 to 0.27, *p* < 0.05) than sense of coherence (r = −0.55 to 0.44, *p* < 0.05), which was correlated to a higher degree with depression (r = −0.58, *p* < 0.05). The relationship between burnout and depression may be a product of the relationship between depression and sense of coherence [83]

### 3.9. Burnout, Job Satisfaction and General Health

One article confirming the relationship between burnout, job satisfaction and health was identified. Job satisfaction was found to be a significant predictor of both burnout and depression, with burnout also significantly predicting depression. Further analysis revealed that job satisfaction moderates the relationship between burnout and health [84]. Further details about the method, sample and findings of identified articles are included in Table 9 below.

**Table 9:** Method, sample and findings of identified articles.

Method	Sample	Findings
Quantitative (questionnaire distribution) [84]	239 nurses in Japan and 550 nurses in mainland China [84]	Job satisfaction among Japanese nurses was found to be a significant predictor of depersonalization (ΔR² = 0.22, *p* < 0.001; β = −0.21, *p* < 0.01), diminished personal accomplishment (ΔR² = 0.10, *p* < 0.001; β = −0.28, *p* < 0.01), and depression (ΔR² = 0.37, *p* < 0.001; β = −0.30, *p* < 0.001). Among Chinese nurses job satisfaction also significantly predicted depersonalization (ΔR² = 0.11, *p* < 0.001; β = −0.12, *p* < 0.05), diminished personal accomplishment (ΔR² = 0.08, *p* < 0.001; β = −0.25, *p* < 0.001), and depression (ΔR² = 0.24, *p* < 0.001; β = −0.18, *p* < 0.001). Emotional exhaustion was found to significantly predict depression in Japanese (ΔR² = 0.37, *p* < 0.001; β = 0.43, *p* < 0.001) as well as Chinese nurses (ΔR² = 0.24, *p* < 0.001; β = 0.38, *p* < 0.001). Absenteeism was not significantly predictive of burnout or job satisfaction. Job satisfaction was found to moderate the relationship between emotional exhaustion and absenteeism in predicting depression among Japanese (ΔR² = 0.03, *p* < 0.01; β = −3.9, *p* < 0.01) and Chinese nurses (ΔR² = 0.02, *p* < 0.05; β = −4.2, *p* < 0.05) [84]

### 3.10. Job Satisfaction and General Health

One article was identified confirming the relationship between job satisfaction and health among nurses by showing that increased job satisfaction was related to poor psychological health among nurses [85]. Further details about the method, sample and findings of identified articles are included in Table 10 below.

**Table 10:** Method, sample and findings of identified articles.

Method	Sample	Findings
Quantitative and qualitative (following a medical pre-examination of mental health as well as interviews about shifts/tasks, questionnaires were distributed to eligible participants) [85]	101 nurses enrolled at a clinic of occupational medicine [85]	Increase in job satisfaction was associated with decreased psychological distress measured using several indicators including perceived stress (r = −0.44, *p* < 0.05) and general health (r = −0.24, *p* < 0.05) scores. Job satisfaction is inversely associated with reduced psychological distress [85]

### 3.11. Work Related Stress, Burnout, Job Satisfaction and General Health

Six articles exploring all variables were identified. As was the case with the findings discussed above, most of these explored two and three way relationships between work related stress and burnout [23,86], work related stress and job satisfaction [23,86,87], work related stress and health [23,86,87,88], burnout job satisfaction and health [89,90] as well as job satisfaction and health [90].

Few of these studies exploring more complex relationships showed that work related stress was not significantly predictive of burnout [90] and only indirectly related to job satisfaction [86]. Additionally, work related stress was found to be a mediator rather than an independent variable predicting burnout, job satisfaction and health among nurses [88].

An additional article identified through a manual search revealed predictive relationships between stressors including information provision, social support, physical conditions and burnout, job satisfaction, somatic complaints respectively. However, the relationship between burnout, job satisfaction and somatic complaints was not empirically explored [91]. Further details about the method, sample and findings of identified articles are included in Table 11 below.

**Table 11:** Method, sample and findings of identified articles.

Method	Sample	Findings
Quantitative (structure questionnaires were mailed with nurses’ paychecks) [23]	173 nurses [23]	Job stress was significantly associated with burnout (r = 0.56, *p* < 0.01) and job satisfaction (r = −0.34, *p* < 0.01). Job stress was significantly associated with psychosomatic health problems (r = 0.55, *p* < 0.01). The only significant interaction was found between job stress and psychosomatic health problems accounting for 5% of the variance (*p* < 0.05) [23]
Quantitative (questionnaire distribution following invitation letters) [86]	1,204 nurses working in general hospitals [86]	The variance explaining job satisfaction was high (*R*² = 0.44). High job satisfaction was significantly (*p* < 0.05) predicted by high social support (β = 0.33), low workload (β = −0.21), low role ambiguity (β = −0.19), low role conflict (β = −0.14) and high autonomy (β = 0.09). Psychosomatic health complaints were explained by high workload (β = 0.20, *p* < 0.05); low social support (β = −0.10, *p* < .05), and high role conflict (β = 0.09, *p* < 0.05). A two-way interaction effect was found between workload and social support (β = −0.08) thereby suggesting that higher levels of social support buffer the negative effects of workload on emotional exhaustion. Results also indicated that high levels of social support would buffer the negative effects of workload on job satisfaction (β = 0.08, *p* < 0.05). High complexity was indirectly predictive of burnout (ΔR² = 0.01, *p* < 0.05; β = −0.08, *p* < 0.05) through mediation by workload (ΔR² = 0.29, *p* < 0.05; β = 0.37, *p* < 0.05) [86]
Quantitative (questionnaires were sent to nurses’ home address) [88]	The sample consisted of 807 registered nurses working in an academic hospital [88]	Organizational and environmental conditions explained significant variance in job characteristics, ranging between 14% in social support colleagues and 41% in workload. Job characteristics explained significant variance in outcomes, ranging between 13% in somatic complaints and 38% in job satisfaction whereas organizational/ environmental conditions explained significant variance in all outcomes: 4% in somatic complaints, 5% in psychological distress, 11% in emotional exhaustion, and 26% in job satisfaction. Occupational stressors overall predict large amounts of the variance in the outcome measures, especially in job satisfaction (44%) and emotional exhaustion (25%). In conclusion, job characteristics (job stressors) mediate the relationship between organisational and environmental conditions and outcomes (burnout, job satisfaction and health) [88]
Quantitative (questionnaire administration) [87]	1,697 registered nurses [87]	Increase in job satisfaction was predicted by emphasis on patient care, recognizing importance of personal lives, satisfaction with salary/benefits, job security and positive relationships with other nurses and managers. Decrease in job satisfaction was predicted by high levels of stress to the point of burnout. Physical health predicted satisfaction with nursing as a career [87]
Quantitative (questionnaire distribution with instructions to return by mail) [89]	175 nurses working in a psychiatric hospital [89]	Job satisfaction was moderately associated with burnout (r = −0.56, *p* < 0.05), which was also moderately associated with psychosomatic health problems (r = 0.45, *p* < 0.05). With shift time as a stressor, significant differences were found in psychosomatic health problems between day and evening shifts (*t* = 2.2, *p* < 0.05), evening and rotational shifts (*t* = −2.3, *p* < 0.05) as well as night and rotational shifts (*t* = −2.10, *p* < .05). For job satisfaction, significant differences were found between day and night shifts (*t* = 2.97, *p* < .05), evening and night shifts (*t* = 2.68, *p* < 0.05) as well as rotational and night shifts (*t* = 3.13, *p* < 0.05). Generally, night shift nurses’ wellbeing seemed to be affected more seriously than nurses working other shifts. Only one interaction effect was found to be significant leading to the conclusion that female nurses on rotating shift experience more health problems than other nurses (F = 3.85, *p* < 0.05) [89]
Quantitative (questionnaire distribution with letter explaining the study) [90]	404 nurses ( 77 male and 317 female) [90]	Job characteristics reflected emotional exhaustion (r = −0.17 to −0.38, *p* < 0.001) but did not explain it. Emotional exhaustion was most highly correlated with job satisfaction (r = −0.55, *p* < 0.001). Both emotional exhaustion (r = 0.25, *p* < 0.001) and job satisfaction (r = −0.12, *p* < 0.05) were related to sickness absence. Job satisfaction was found to be a strong predictor of emotional exhaustion (β = −0.42, *p* = 0.001). The most prominent predictor of sickness absence was emotional exhaustion (β = 0.29, *p* = 0.001) [90]
Quantitative (questionnaire distribution by the matron and researchers in each ward) [91]	309 female nurses working in private and public hospitals in 3 countries [91]	Burnout is most strongly predicted by problems with information provision (ΔR² = 0.17, *p* < 0.001; β = −0.20, *p* < 0.001), job satisfaction by lack of social support form supervisors (ΔR² = 0.36, *p* < 0.001; β = 0.21, *p* < 0.001) and somatic complaints by physical working conditions (ΔR² = 0.08, *p* < 0.01; β = 0.16, *p* < 0.05) [91]

## 4. Discussion

The majority of the articles included in this review have revealed that high levels of work related stress, burnout, job dissatisfaction and poor health are common within the nursing profession. This is supported by literature suggesting that nurses experience longer working hours as well as frequent direct, personal and emotional contact with a large number of patients in comparison with other health professionals [10,91].

Although a number of articles identified in this review have confirmed significant relationships between work related stressors and burnout [25,26,27,28,29,30,31,32,33,34,59,60,77,78], job satisfaction [11,36,37,38,39,40,41,42,43,44,45,46,47,48,49,50,51,58,59,60,61,62,63,64,65,66,81] as well as general health, these relationships are predominantly two way relationships with only a handful of studies confirming three way relationships [64,65,71,84]. Among the studies confirming two way relationships, only one study confirming the relationship between job satisfaction and general health [85] was identified. Similarly, only one study confirming the three way relationship between burnout, job satisfaction and general health [84] was identified. This demonstrates the limited availability of studies exploring certain relationships.

Among the handful of studies confirming three way relationships findings suggest that work related stress significantly predicts burnout, which is significantly predictive of physical and mental health symptoms. This means that burnout plays an intervening role in the relationship between work related stress and general health among nurses [71]. Although such findings provide strong support for the relationship between work-related stress, burnout and general health, little is known about the role of job satisfaction. Within the literature, ample evidence confirming significant two way relationships between work related stress and job satisfaction [49,50,51], burnout and job satisfaction [81] as well as general health and job satisfaction [78,85] is available. However, limited evidence to account for mediation and moderation in the relationship between all variables could be found.

Furthermore, within studies confirming three way relationships, available evidence regarding the role of job satisfaction is conflicting in that one study reveals that job satisfaction is a significant predictor of burnout among nurses, [11] whereas another study reveals that job satisfaction is the outcome variable predicted by work related stress and burnout [64]. Contradictory to this, it has also been found that job satisfaction is the intervening variable in the relationship between burnout and general health [84]. Therefore, despite work related stress, burnout, job satisfaction and general health being inter-related, the complexity of these relationships can only be well understood if all variables are explored simultaneously.

### 4.1. Limitations

Limitations of the studies included in this review involve predominant exploration of two and three way relationships between work related stress, burnout, job satisfaction and general health of nurses, while focusing less on the relationship between all four variables. Furthermore, majority of the studies included in this review have used cross-sectional study designs with only a few longitudinal studies; hence the evidence base for causality is still limited. As such, there is minimal evidence supporting the causal nature of relationships between all four variables. Moreover, the use of different measuring instruments, biased samples and in some cases poor response rates compromise the generalisability of findings. Limitations of the review with regards to the inclusion of studies published only in English, introduces a language bias. Additionally, most studies included in this review were conducted in developed countries, thereby limiting generalizability to nurses in developing countries. 

### 4.2. Implications

Comprehensive review of all variables, revealed some contradictory evidence for the role of job satisfaction in the relationship between work related stress, burnout and general health, indicating the need for further research confirming the role of job satisfaction. Although it was found that the nature and direction of relationships between these variables is ambiguous, identification of this gap in findings emphasizes the importance of simultaneously exploring the relationship between all four variables towards understanding causality.

## 5. Conclusions

Identified relationships in this review were mostly two- and to a lesser extent three-way relationships, with minimal focus on the causal nature and direction of relationships. Further research exploring mediating and moderating effects of relationships between work related stress, burnout, job satisfaction and general health over longer periods of time are necessary for establishing causality. Understanding causality will allow for specific and appropriate strategies to address challenges of work related stress, burnout, job dissatisfaction and poor general health among nurses, such as low productivity, poor service delivery and adverse patient outcomes [92,93].

## References

[1] Maslach C., Jackson S.E. (1981). The measurement of experienced burnout. J. Occup. Behav..

[2] Maslach C., Jackson S.E. (1986). Maslach Burnout Inventory: Manual.

[3] Vanyperen N.W., Buunk B.P., Schaufeli W.B. (1992). Communal orientation and the burnout syndrome among nurses. J. Appl. Soc. Psychol..

[4] Maslach C. (2003). Burnout: The Cost of Caring.

[5] Chopra S.S., Sotile W.M., Sotile M.O. (2004). Physician burnout. J. Am. Med. Assoc..

[6] Rada R.E, Johnson-Leong C. (2004). Stress, burnout, anxiety and depression among dentists. J. Am. Dent. Assoc..

[7] Aiken L.H., Clarke S.P., Sloane D.M., Sochalski J., Silber J. (2002). Hospital nurse staffing and patient mortality, nurse burnout, and job dissatisfaction. J. Am. Med. Assoc..

[8] Engelbrecht M., Bester C.L., Van Den Berg H., Van Rensburg H.C.J. The Prediction of Psychological Burnout by Means of the Availability of Resources, Time Pressure or Workload, Conflict and Social Relations and Locus of Control of Professional Nurses in Public Health Centres in the Free State. proceedings of the European Applied Business Conference (EABR) and Teaching and Learning Conference (TLC).

[9] Jennings B.M., Hughes R.G. (2008). Work Stress and Burnout among Nurses: Role of the Work Environment and Working Conditions. Patient Safety and Quality: An Evidence Based Handbook for Nurses.

[10] Levert T., Lucas M., Ortlepp K. (2000). Burnout in psychiatric nurses: Contributions of the work environment and a sense of coherence. S. Afr. J. Psychol..

[11] Kalliath T., Morris R. (2002). Job satisfaction among nurses: A predictor of burnout levels. J. Nurs. Admin..

[12] Turnbull J.M., Hamdy R.C., Turnbull J.M., Clark. W, Lancaster M.M. (1994). Stress in Nursing Care. Alzheimer’s Disease: A Handbook for Caregivers.

[13] Demeuroti F., Bakker A.R., Nachreiner F., Schaufeli W.B. (2000). A model of burnout and life satisfaction among nurses. J. Adv. Nurs..

[14] Lim J., Bogossian F., Ahern K. (2010). Stress and coping in Australian nurses: A systematic review. Int. Nurs. Rev..

[15] Nolan P., Smojkis M. (2003). The mental health of nurses in the UK. Adv. Psychiatr. Treat..

[16] Coyle D., Edwards D., Hannigan B., Fothergill A., Burnard P. (2005). A systematic review of stress among mental health social workers. Int. Soc. Work.

[17] Kohler J.M., Munz D.C., Grawitch M.J. (2006). Test of a dynamic stress model for organisational change: Do males and females require different models?. Appl. Psychol. Int. Rev..

[18] Peltzer K., Mashego T., Mabeba M. (2003). Short communication: Occupational stress and burnout among South African medical practitioners. Stress Health.

[19] Rothmann S., Van Der Colff J.J., Rothmann J.C. (2006). Occupational stress of nurses in South Africa. Curationis.

[20] Lu H., Barriball K.L., Zhang X, While A.E. (2012). Job satisfaction among hospital nurses revisited: A systematic review. Int. J. Nurs. Stud..

[21] Toh S.G., Ang E., Devi M.K. (2012). Systematic review on the relationship between the nursing shortage and job satisfaction, stress and burnout levels among nurses in oncology/haematology settings. Int. J. Evid. Based Healthc..

[22] O’Mahony N. (2011). Nurse burnout and the working environment. Emerg. Nurse.

[23] Jamal M., Baba V.V. (2000). Job stress and burnout among Canadian managers and nurses: An empirical examination. C. J. Public Health.

[24] Goldberg D.P., Hillier V.F. (1979). A scaled version of the General Health Questionnaire. Psychol. Med..

[25] Chayu T., Kreitler S. (2011). Burnout in nephrology nurses in Israel. Nephrol. Nurs. J..

[26] Lee J.S.Y., Akhtar S. (2011). Effects of the workplace social context and job content on nurse burnout. Hum. Resource Manage..

[27] Ohue T., Moriyama M., Nakaya T. (2011). Examination of a cognitive model of stress, burnout and intention to resign for Japanese nurses. Jpn. J. Nurs. Sci..

[28] Melchior M.E.W., Van Den Berg A.A., Halfens R., Huyer Abu-Saad H., Philipsen H., Gassman P. (1997). Burnout and the work environment of nurses in psychiatric long-stay care settings. Soc. Psych. Psych. Epid..

[29] Hayter M. (1999). Burnout and AIDS care related factors in HIV community nurse specialists. J. Adv. Nurs..

[30] Garrosa E., Rainho C., Moreno-Jimenez B., Monteiro M.J. (2010). The relationship between job stressors, hardy personality, coping resources and burnout in a sample ofnurses: A correlational study at two time points. Int. J. Nurs. Stud..

[31] Gueritault-Chalvin V., Kalichman S.C., Demi A., Peterson J.L. (2000). Work-related stress and occupational burnout in AIDS caregivers: Test of a coping model with nurses providing AIDS care. AIDS Care.

[32] Patrick K., Lavery J.F. (2007). Burnout in nursing. Aust. J. Adv. Nurs..

[33] Wu S., Zhu W., Wang Z., Wang M., Lan Y. (2007). Relationship between burnout and occupational stress among nurses in China. J. Adv. Nurs..

[34] Barnard D., Street A., Love A.W. (2006). Relationships between stressors, work supports and burnout among cancer nurses. Cancer Nurs..

[35] Lasebikan V.O., Oyetunde M.O. (2012). Burnout among nurses in a Nigerian general hospital: Prevalence and associated factors. ISRN Nurs..

[36] Archibald C. (2006). Job satisfaction among neonatal nurses. Pediatr. Nurs..

[37] Ernst M., Franco M., Messmer P.R., Gonzalez J.L. (2004). Practice applications of research. Nurses’ job satisfaction, stress and recognition in a pediatric setting. Pediatr. Nurs..

[38] Penz K., Stewart N.J., D’Arcy C., Morgan D. (2008). Predictors of job satisfaction for rural acute care registered nurses in Canada. Western J. Nurs. Res..

[39] Applebaum A., Fowler S., Fiedler N., Osinubi O., Robson M. (2010). The impact of environmental factors on nursing stress, job satisfaction and turnover intention. J. Nurs. Admin..

[40] Chaboyer W., Williams G., Corkill W., Creamer J. (1999). Predictors of job satisfaction in remote hospital nursing. Can. J. Nurs. Leadersh..

[41] Djukic M., Kovner C., Budin W.C., Norman R. (2010). Physical work environment: Testing an expanded model of job satisfaction in a sample of registered nurses. Nurs. Res..

[42] Iliopoulou K.K., While A.E. (2010). Professional autonomy and job satisfaction: Survey of critical care nurses in mainland Greece. J. Adv. Nurs..

[43] Malliarou M., Sarafis P., Moustaka E., Kouvela T., Constantinidis T.C. (2010). Greek registered nurses’ job satisfaction in relation to work-related stress. A study on army and civilian RNs. Global J. Health Sci..

[44] Graham K., Davies B., Woodend K., Simpson J., Mantha S. (2011). Impacting Canadian public health nurses’ job satisfaction. C. J. Public Health.

[45] Healy C., McKay M.F. (2000). Nursing stress: The effects of coping strategies and job satisfaction in a sample of Australian nurses. J. Adv. Nurs..

[46] Zurmehly J. (2008). The relationship of educational preparation, autonomy, and critical thinking to nursing job satisfaction. J. Contin. Educ. Nurs..

[47] Wanzer M., Wojtaszczyk A.M., Kelly J. (2009). Nurses’ perceptions of physicians’ communication: The relationship among communication practices, satisfaction and collaboration. Health Commun..

[48] Ho W.H., Chang C.S., Shih Y.L., Liang R.D. (2009). Effects of job rotation and role stress among nurses on job satisfaction and organizational commitment. BMC Health Serv. Res..

[49] Flanagan N.A., Flanagan T.J. (2002). An analysis of the relationship between job satisfaction and job stress in correctional nurses. Res. Nurs. Health.

[50] Larrabee J.H., Wu Y., Persily C.A., Simoni P.S., Johnston P.A., Marcischak T.L., Mott C.L., Gladden S.D. (2010). Influence of stress resiliency on RN job satisfaction and intent to stay. Western J. Nurs. Res..

[51] Cangelosi J., Markham F., Bounds W. (1998). Factors related to nurse retention and turnover: An updated study. Health Mark. Q..

[52] Piko B.F. (2003). Psychosocial work environment and psychosomatic health of nurses in Hungary. WorkStress.

[53] Lin K.C., Huang C.C., Wu C.C. (2007). Association between stress at work and primary headache among nursing staff in Taiwan. Headache.

[54] Malinauskienė V., Leišytė P., Malinauskas R. (2009). Psychosocial job characteristics, social support, and sense of coherence as determinants of mental health among nurses. Med. Lith..

[55] Lambert V.A., Lambert C.E., Petrini M., Li X.M., Zhang Y.J. (2007). Predictors of physical and mental health in hospital nurses within the People’s Republic of China. Int. Nurs. Rev..

[56] Boey K.W., Chan K.B., Ko Y.C., Goh C.G., Lim G.C. (1997). Work stress and psychological well-being among nursing professionals in Singapore. Singap. Med. J..

[57] Tucker S.J., Harris M.R., Pipe T.B., Stevens S.R. (2010). Nurses’ ratings of their health and professional work environments. Am. Assoc. Occ. Health Nurses J..

[58] McHugh M.D., Kutney-Lee A., Cimiotti J., Sloane D.M., Aiken L.H. (2011). Nurses’ widespread job dissatisfaction, burnout, and frustration with health benefits signal problems for patient care. Health Affair..

[59] Liu K., You L.M., Chen S.X., Hao Y.T., Zhu X.W., Zhang L.F., Aiken L.H. (2012). The relationship between hospital work environment and nurse outcomes in Guangdong, China: A nurse questionnaire survey. J. Clin. Nurs..

[60] Kiekkas P., Spyratos F., Lampa E., Aretha D., Sakellaropoulos G.C. (2010). Level and correlates of burnout among orthopaedic nurses in Greece. Orthop. Nurs..

[61] Cho S.H., June K.J., Kim Y.M., Cho Y.A., Yoo C.S., Yun S.C., Sung Y.H. (2009). Nurse staffing, quality of nursing care and nurse job outcomes in intensive care units. J. Clin. Nurs..

[62] Sheward L., Hunt J., Hagen S., Macleod M., Ball J. (2005). The relationship between UK hospital nurse staffing and emotional exhaustion and job dissatisfaction. J. Nurs. Manage..

[63] Bogaert P.V., Meulemans H., Clarke S., Vermeyen K., van de Heyning P. (2009). Hospital nurse practice environment, burnout, job outcomes and quality of care: Test of a structural equation model. J. Adv. Nurs..

[64] Bogaert P.V., Clarke S., Vermeyen K., Meulemans H., van de Heyning P. (2009). Practice environments and their associations with nurse-reported outcomes in Belgian hospitals: Development and preliminary validation of a Dutch adaptation of the Revised Nursing Work Index. Int. J. Nurs. Stud..

[65] Bogaert P.V., Clarke S., Roelant E., Meulemans H., van de Heyning P. (2010). Impacts of unit-level nurse practice environment and burnout on nurse-reported outcomes: A multilevel modelling approach. J. Clin. Nurs..

[66] Klopper H.C., Coetzee S.K., Pretorius R., Bester P. (2012). Practice environment, job satisfaction and burnout of critical care nurses in South Africa. J. Nurs. Manage..

[67] van Servellen G., Topf M., Leake B. (1994). Personality hardiness, work-related stress, and health in hospital nurses. Hosp. Top..

[68] van Tooren M., de Jonge J. (2008). Managing job stress in nursing: What kind of resources do we need?. J. Adv. Nurs..

[69] Jourdain G., Chenevert D. (2010). Job demands-resources, burnout and intention to leave the nursing profession: A questionnaire study. Int. J. Nurs. Stud..

[70] Bressi C., Manenti S., Porcellana M., Cevales D., Farina L., Felicioni I., Meloni G., Milone G., Miccolis I.R., Pavanetto M. (2008). Haemato-oncology and burnout: An Italian survey. Brit. J. Cancer.

[71] Hillhouse J.J., Adler C. (1996). Evaluating a simple model of work, stress, burnout, affective and physical symptoms in nurses. Psychol. Health Med..

[72] Sortet J., Banks S. (1996). Hardiness, job stress and health in nurses. Hosp. Top..

[73] Bourbonnais R., Comeau M., Vézina M., Dion G. (1998). Job strain, psychological distress, and burnout in nurses. Am. J. Ind. Med..

[74] Baglioni A.J., Cooper C.L., Hingley P. (1990). Job stress, mental health and job satisfaction among U.K. senior nurses. Stress Med..

[75] Landerweerd J.A., Boumans N.P.G. (1994). The effect of work dimensions and need for autonomy on nurses’ work satisfaction and health. J. Occup. Organ. Psych..

[76] Tummers G.E.R., Landeweerd J.A., Merode G.V. (2002). Organization, work and work reactions: A study of the relationship between organizational aspects of nursing and nurses’ work characteristics and work reactions. Scand. J. Caring Sci..

[77] Decker F.H. (1997). Occupational and non occupational factors in job satisfaction and psychological distress among nurses. Res. Nurs. Health.

[78] Engström M., Ljunggren B., Lindqvist R., Carlsson M. (2006). Staff satisfaction with work, perceived quality of care and stress in elderly care: Psychometric assessments and associations. J. Nurs. Manage..

[79] Piko B.F. (1999). Work related stress among nurses: A challenge for health care institutions. J. R. Soc. Promo. Health.

[80] Adriaenssens J., Gucht V.M.J., Doef M.P., Maes S. (2011). Exploring the burden of emergency care: Predictors of stress-health outcomes in emergency nurses. J. Adv. Nurs..

[81] Önder C., Basim N. (2008). Examination of developmental models of occupational burnout using burnout profiles of nurses. J. Adv. Nurs..

[82] Iacovides A., Fountoulakis K.N., Moysidou C., Ierodiakonou C. (1999). Burnout in nursing staff: Is there a relationship between depression and burnout?. Int. J. Psychiat. Med..

[83] Tselebis A., Moulou A., Ilias I. (2001). Burnout *versus* depression and sense of coherence: Study of Greek nursing staff. Nurs. Health Sci..

[84] Tourigny L., Baba V.V., Wang X. (2010). Burnout and depression among nurses in Japan and China: The moderating effects of job satisfaction and absence. Int. J. Hum. Resour. Man..

[85] Amati M., Tomasetti M., Ciuccarelli M., Mariotti L., Tarquini L.M., Bracci M., Baldassari M., Balducci C., Alleva R., Borghi B. (2010). Relationship of job satisfaction, psychological distress and stress-related biological parameters among healthy nurses: A longitudinal study. J. Occup. Health.

[86] Tummers G.E.R., Landeweerd J.A., van Merode G.G. (2002). Work organization, work characteristics and their psychological effects on nurses in the Netherlands. Int. J. Stress. Manage..

[87] Buerhaus P.I., Donelan K., Ulrich B.T., Kirby L., Norman L., Dittus R. (2005). Registered nurses’ perceptions of nursing. Nurs. Econ..

[88] Gelsema T.I., van Doef M., Maes S., Akerboom S., Verhoeven C. (2005). Job stress in the nursing profession: The influence of organizational and environmental conditions and job characteristics. Int. J. Stress Manage..

[89] Jamal M., Baba V.V. (1997). Shiftwork, burnout and well-being: A study of Canadian nurses. Int. J. Stress Manage..

[90] Bekker M.H.J., Croon M.A., Bressers B. (2005). Childcare involvement, job characteristics, gender and work attitudes as predictors of emotional exhaustion and sickness absence. Work Stress.

[91] Van Der Doef M., Mbazzi F.B., Verhoeven C. (2012). Job conditions, job satisfaction, somatic complaint and burnout among East African nurses. J. Clin. Nurs..

[92] Maslach C., Leiter M.P. (1997). The Truth About Burnout: How Organisations Cause Personal Stress and What To Do About It.

[93] Hickman D., Severance S., Feldstein A. (2003). The Effect of Health Care Working Conditions on Patient Safety.

